# Surface morphology and chemical composition analysis of titanium dental implants using SEM and EDX

**DOI:** 10.6026/973206300200926

**Published:** 2024-08-31

**Authors:** Mohammed Mathar

**Affiliations:** 1 Department of Prosthetic Dental Sciences, College of Dentistry, Qassim University, KSA

**Keywords:** Dental implants, Scanning Electron Microscopy, Energy Dispersive X-ray spectroscopy, Surface morphology and chemical composition

## Abstract

The chemical content and surface morphology of titanium implants have a greater impact on the osseointegration characteristic of dental implants. Therefore this study was done to examine the surface morphology and chemical composition of commercially available titanium dental implants. (BioLine Dental Implants Series-single piece (A) and spiral implants (B)). The chemical composition was determined by the Energy Dispersive X-ray Spectroscopy (EDX) method and the surface morphology was performed by Scanning Electron Microscopy (SEM) method. The results for chemical composition of titanium implants using EDX method revealed that titanium (Ti) constitutes the major surface components on the plain and top land area of the single piece compressive implants which is 89.71 weight% and 71.55 weight % respectively. Iron (Fe)-66.60 weight % is considered as major element along with chromium (Cr)-18.34 weight %on the plain area of spiral implants and O (19.90 weight %) and Al (12.43 weight %) on the top land area of spiral implants. The surface morphology and impact of the manufacturing process on the implant surface using SEM method revealed that the similar surface irregularities with diameter ranging from 10 µm to 20 µm on the side view of the top land area and diameter of 10 µm on the side view of plain area for both the samples. Sample A showed some amorphous structures with grainy marks on the apical view whereas Sample B showed heavy grinding and shear marks on the apical view at diameter ranging from 10 µm to 20µm.

## Background:

Osseointegration is the process of a direct contact between an implant and bone without disrupting the soft tissue layer [1]. Continuous research is conducted to create newer designs that will have greater clinical success than the implant systems that are presently available. Physical, chemical, or mechanical methods can be used to alter the surface morphology to enhance osseointegration. Surface properties can be classified into mechanical, topographic and physiochemical properties. The surface roughness is an essential component of surface topography, enhancing osseointegration [14]. In the surface properties of dental implants, topographic and physiochemical changes can be employed to improve osseointegration and primary implant stability [2]. Therefore, detailed surface characterization is important to better understand implant integration in bone through surface properties. Due to their advantageous combination of characteristics including high corrosion resistance, excellent biocompatibility, low specific weight and low modulus of elasticity, titanium and its alloys are incredibly successful materials for the construction of dental and orthopedic implants [3].

When titanium (Ti), a reactive material, comes into contact with air or water, a nano-thick layer of titanium oxide (TiO2) develops, improving the implant's biocompatibility, compatibility with the material's bone interface, and corrosion resistance [4]. According to several in vivo studies, surface chemistry of titanium implants is crucial for osseointegration. Recently, osseointegration was given a new definition "osseointegration is a foreign body reaction where interfacial bone is formed as a defense reaction to shield off the implant from the tissues" [5,6]. It is well known that surface microstructure or roughness plays a significant role in the interactions between the cell and the tissue during the osseointegration process Titanium implants in particular are known to interact with bone tissue, resulting in a significant portion of the implant surface coming into close contact with the newly formed bone tissue which is more important for stability and success of implants [7].

## The surface characteristics of titanium implants due to its excellent biocompatibility and osseointegration includes:

[1] A dense, extremely durable inactive oxide layer that shields the underlying metal from further oxidation and corrosion.

[2] A very low concentration of charged titanium corrosion products and a slow rate of oxide layer dissolution. Since corrosion and ion release into the surrounding tissue are undesirable, the thickness and stability of the oxide film are important to implant function.

[3] Depending on the chemical and topographic characteristics of the surface, an acceptable biological reaction across the entire spectrum of interactions between water, proteins and cells can be achieved.

[4] When implanted directly without using cement, the material has a strong ability to osseointegrated resulting in a high proportion of direct bone contact [8, 9].

The current research was aimed to analyze the surface morphology and chemical composition of commercially available dental implant system (Bioline Dental Implants Series-single piece and spiral implants). Surface morphology analysis was conducted using scanning electron microscopy (SEM), and depth profile measurements using energy dispersive X-ray spectroscopy (EDX) were used to identify the unique elements of the implants.

## Materials and Methods:

## Description of Implants system:

Single piece Bioline Dental Implant Series (3.75 x 10 mm, Bioline dental GmbH & Co.KG, Berlin, Germany) (Sample A) and Spiral Bioline Dental Implants Series (3.75 x 10 mm, Bioline dental GmbH & Co.KG, Berlin, Germany) (Sample B) were used in this study. Single piece implant is a single piece solid designed implant with a build in abutment on top and has active sharp threaded implants along with build in abutment allow us to get initial stability during immediate loading implant placement even in extreme atrophic ridges. The advantages of single piece implants series are there is no connection between solid strong neck & abutment, bendable neck for perfect angulation compensation and parallelism, aggressive design for better primary stability, having wide range of designs, diameters and length with surface treatment options and keyhole flapless procedure can be performed.

Single piece implant series are classified into three types as compressive, basal, and zygomatic implant. In the present study, single piece compressive implant was used. The single piece compressive implant is designed in such a way that it compresses and condenses the natural native bone due under drilling protocol and to preserve the bone. The advantages of single piece compressive implants are user friendly especially for narrow ridges, adequate build in platform switching for soft tissue growth, smooth antibacterial collar prevents peri-implantitis, self-tapping for easy insertion and is highly recommended for immediate placement and loading techniques.

Spiral Bioline dental implants are designed as tapered threaded implant and have dynamic self-drilling capability. The advantages of spiral implants are user friendly, higher bone to implant contact leads to excellent primary stability which in turn reduces bone resorption. The anodized coating of spiral implants helps maintain internal connections between implant restoration parts, increases mechanical strength, reduces friction between bare metal, and guarantees long-term success of rehabilitation on implants.

## SEM analysis:

SEM (EVO 18, Carl Zeiss Microscopy GmbH, and Germany) was used to perform the surface morphology of two implants (Sample A & Sample B). The SE mode has an acceleration voltage of 20 kV, a magnification range of 1X to 250KX, and a working distance of 9-16 mm. 200 pA of beam current was applied. SEM images of the coronal, middle, and apical regions of each implant were taken. The samples were positioned on the carbon plates inside the vacuum-sealed microscopic chamber.

## EDX analysis:

Analytical or chemical elements can be characterized using the scientific method known as energy dispersive X-ray spectroscopy (EDX). EDX equipment is usually attached to an electron microscope, such as a transmission electron microscope (TEM) or scanning electron microscope (SEM). The unique X-rays that are released from a specimen form the basis of EDX. A stream of high-energy charged particles (electrons or protons) aims for the intended sample. An X-ray with energy comparable to the dissimilarity among the electron level binding energies is released when an electron from a level with a higher electron binding energy penetrates the core hole. The peaks connected to the substance under investigation's elemental composition can be seen in a spectrum generated by EDX analysis. The elemental mapping of a sample can be created using this characterization method [10].

EDX is a commonly used method for determining and calculating the elemental composition of a very small sample of material when a scanning electron microscope (SEM) is configured properly, the electron beam excites the surface atoms, causing them to release a variety of X-rays at specific wavelengths that reveal the atomic structure of the elements. An energy dispersive detector, a solid-state device that can differentiate between X-ray energies can analyze this X-ray radiation. By allocating the appropriate elements, the composition of the atoms on the object surface is established. This method is referred to as energy dispersive X-ray spectroscopy (EDX), is useful for determining the composition of the surface of a specimen. Energy dispersive X-ray spectroscopic (EDX) analysis was performed to determine the elemental composition of two implants of Sample A & Sample B.

## Results:

## SEM analysis of single piece compressive implant (Sample A):

**Figure 1:**
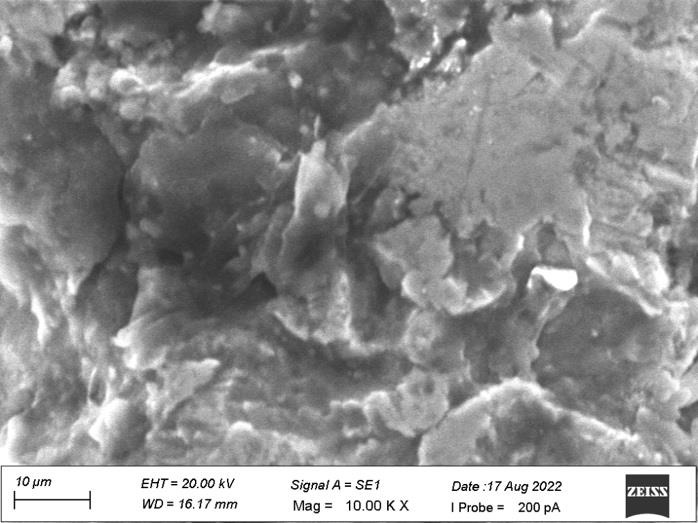
No grain formation on the side view of the top land area.

**Figure 2:**
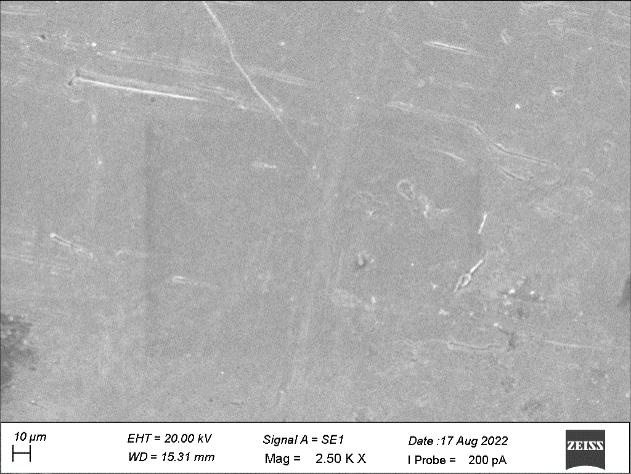
Dimple surface on the side view of the plain area

**Figure 3:**
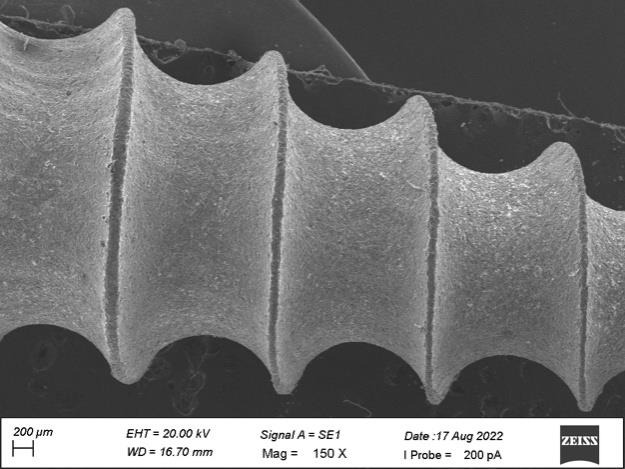
Micro thread patterns with non-grainy surface at the middle level of the side view

**Figure 4:**
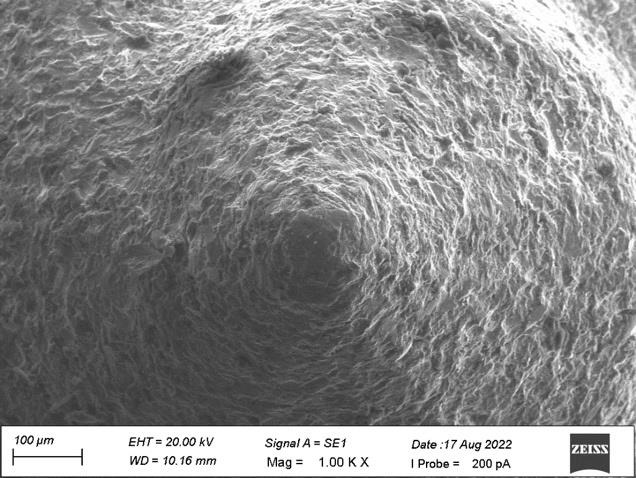
Grinding lines with grainy marks on the apical view

**Figure 5:**
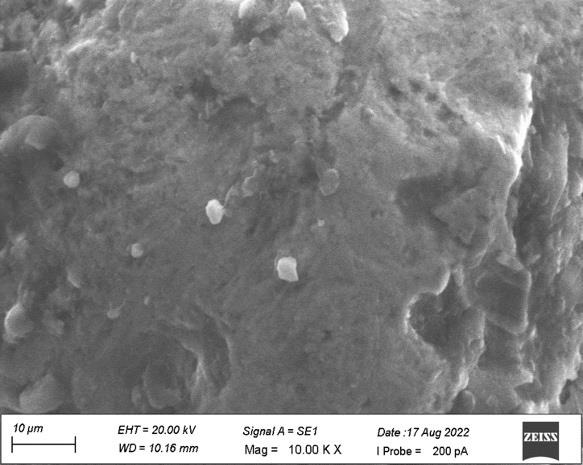
Some amorphous structures with grainy marks on the apical view

Figure 1 revealed the surface irregularities on the side view of the top land area with diameter ranging from 10 µm to 20 µm which shows no grain formation. Figure 2 shows some dimple surfaces and scratch marks at a diameter of 10 µm on the side view of plain area. Figure 3 revealed the micro thread patterns were distributed uniformly with non-grainy surface with diameter 200 µm at the middle level on the side view. Figure 4 showed grinding lines with grainy marks and improper surface morphology with diameter ranging from 100 µm to 200 µm on the apical view. Figure 5 denoted some amorphous structures with grainy marks at diameter ranging from 10 µm to 20 µm on the apical view.

## SEM analysis of spiral implants (Sample B):

**Figure 6:**
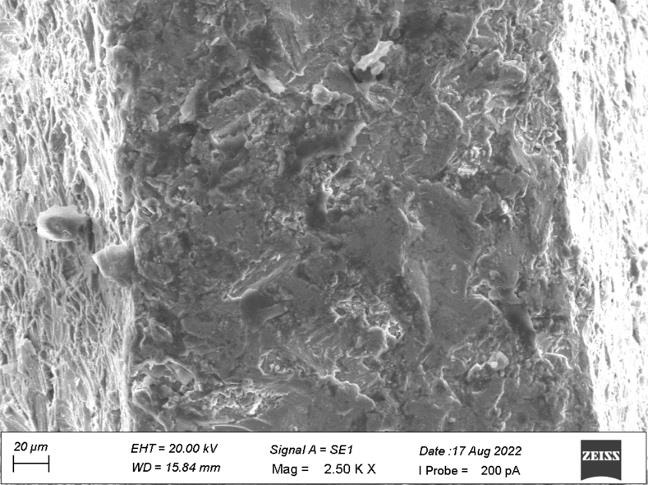
Surface irregularities on the side view of the top land area

**Figure 7:**
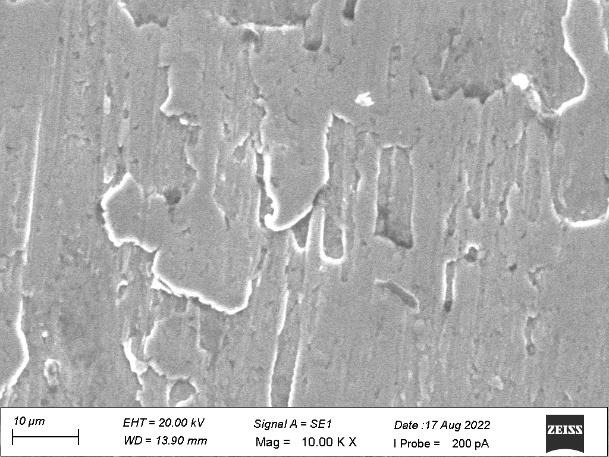
Heavy grinding surface and shear marks on the side view of the plain area

**Figure 8:**
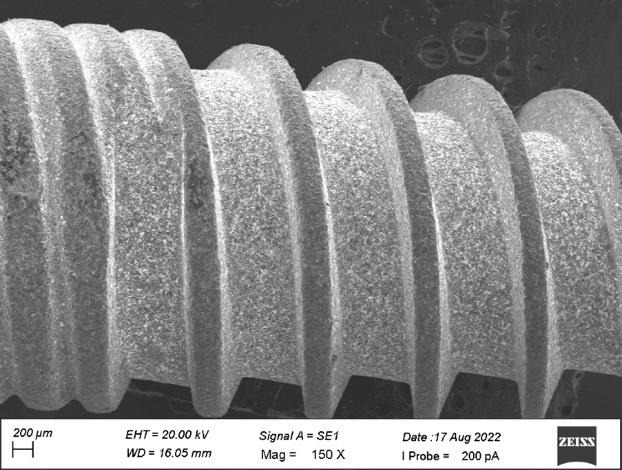
Surface irregularities and improper morphology with flat tip on the apical view

**Figure 9:**
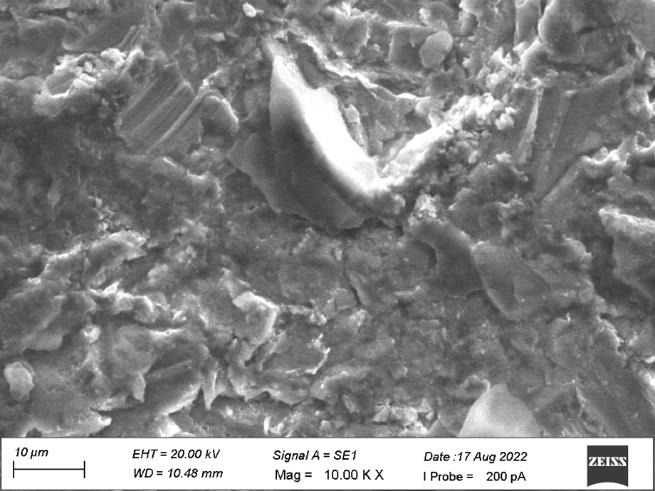
Heavy grinding and shear marks on the apical view

Figure 6 revealed surface irregularities with diameter ranging from 10 µM to 20 µM on the side view of the top land area. Figure 7 showed the heavy grinding surface and shear marks at 10µm diameters on the side view of the plain area. Figure 8 denoted surface irregularities and improper morphology with flat tip on the apical view of implant with diameter ranging from 100µM to 200µM Figure 9 showed heavy grinding and shear marks with diameter ranging from 10 µM to 20µM on the apical view of spiral implant.

## EDX analysis of single piece compressive implant (Sample A):

**Figure 10:**
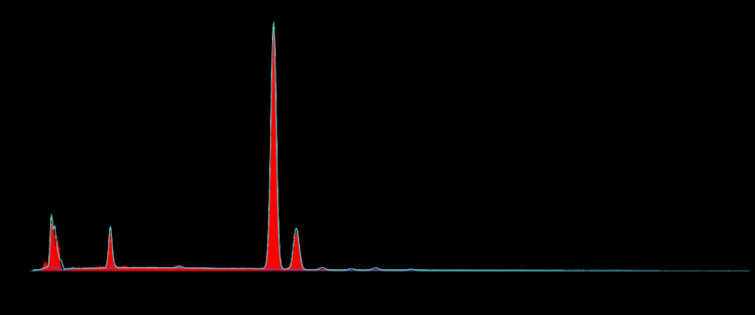
Elemental composition on the plain area of single piece compressive implant (Sample A)

According to the weight%, the elemental composition on the plain area and top land area were analyzed and the results were shown in Table 1, Table 2, Table 3. Figure 10 and Table 1 indicate variations in reading for elemental composition on the plain and top land area for sample A.

## EDX analysis of spiral implants (Sample B):

According to the weight%, the elemental composition on the plain area and top land area were analyzed and the results were observed in Table 2 & Table 3.

## Discussion:

The macroscopic design of the implant permits the primary stability needed for the implant's biological process, and the surface characteristics of the implant are the two main factors that contribute to the stability and responsiveness of implants to produce effective outcomes [1]. Numerous researches have examined how rough surfaces affect cell aggregation and the development of the oxide layer. On the other hand, the oxide layer did not exhibit any changes. A crucial element in the osseointegration process is micro surface alteration of the implant surface's outermost atomic layer. The biocompatibility and prognosis of osseointegration of implants can be significantly impacted by surface and compositional atomic-level changes on the implant surface [13].

The three main types of surface characteristics are mechanical, topographic, and physiochemical. To enhance osseointegration and primary implant stability, topographic and physiochemical alterations can be made to the surface features of dental implant. The surface roughness profile of titanium implants can have an impact on the effectiveness of osseointegration and biomechanical fixation [14]. The adhesion, proliferation, and differentiation of cells are all known to be improved by increased surface roughness [15]. The geometry of the implant surface affects the expression of extracellular matrix proteins, osteoblast differentiation, and proliferation [15].

SEM study of these implant systems revealed the surface morphology and effect of manufacturing process on implant surface. For the purpose of analyzing the elemental compositions of samples of organic and inorganic material, EDX is a standard technique that is widely employed in dental research [11]. Nearly all implants had carbon residuals on their surface, according to the EDX analysis, which is consistent with the majority of investigations [11]. Implant features including thickness, chemical composition, and microstructure in the oxide layer of the final product to be marketed will be influenced by a number of parameters, including pressure, machining velocity, surface treatment, cleaning, sterilization, packaging, and storage. To enhance the alloy's mechanics and physical/chemical behaviour, some components have been added. Nevertheless, these can cause contamination of the last oxide layer on the surface, which could alter the behaviour of the cell either favorably or unfavorably [15].

Different reactions will be encouraged in the surrounding media by the chemical makeup. SEM analysis of these implants systems showed the surface morphology and effects of the manufacturing process on the implant surface. The morphology, chemical content, and topography of dental implants with a moderate level of roughness were assessed by Fahlstedt *et al.* On every implant surface, they discovered changed surface topography and chemical composition [16].

In the present study, both the samples revealed the similar surface irregularities with diameter ranging from 10µm to 20µm on the side view of the top land area and diameter of 10µm on the side view of plain area. Sample A showed some dimple surfaces whereas sample B revealed heavy grinding surfaces. The micro thread patterns for both the samples showed the uniform distribution at the middle level of the thread plank at 200µm diameters. Sample A showed some amorphous structures with grainy marks on the apical view whereas Sample B showed heavy grinding and shear marks on the apical view at diameter ranging from 10µm to 20 µM.

In order to enhance the clinical performance of implants and produce a robust mechanical bone-implant interface, a number of techniques have been developed to create rough implant surfaces. Dental implant titanium surfaces can also be roughened by etching with powerful acids like HCl, H2SO4, HNO3, and HF [15].

Surfaces treated with HCl-HF-H3PO4 exhibited superior biocompatibility, reduced cytotoxicity, and increased roughness in comparison to control samples, as demonstrated by Zareidoost *et al.* Furthermore, a significant, popular, and novel method for producing bio function in metals for biomedical applications, including dentistry, is the addition of calcium chloride to a mixed solution of three acids that contains HCl, HF, and H3PO4 [15].

Before and after photo functionalization, the surface morphology and elemental composition of zirconia implants are analyzed by Jaikumar *et al.* They came to the conclusion that photo functionalization is a workable way to improve the surface topography of zirconia implants. The biocompatibility and prognosis of osseointegration of implants can be significantly impacted by surface and compositional atomic-level changes on the implant surface [13].

In present study, two commercially available clinically successful dental implants were examined in vitro to assess their surface properties. Ti, O and C have been identified as the main elements in previous studies on the surface chemistry of implants that have been machined and blasted. In conjunction with the applied techniques, the chemically changed implants have shown more complex compositions involving Ca, P, Mg, S, F, and Na. In the present study of EDX analysis, Tiis the major surface component on the plain and top land area of the single piece compressive implants which is 89.71 weight% and 71.55 weight % respectively along with Al consists of 7.32 weight % on the plain area and 18.86 weight % on the top land area of single piece compressive implants suggestive of Ti-Al alloy (Sample A). Fe (66.60 weight %) is considered as major element along with Cr (18.34 weight %) on the plain area of spiral implants and O (19.90 weight %) & Al (12.43 weight %) on the top land area of spiral implants (Sample B).

José Dias *et al.* came to the conclusion that, even if the majority of the examined samples had identical implant shape, over 50% of them-that is, brands of implants that are sold on the market-showed aluminum on the implant surface. Lastly, it can be said that, of the samples examined, STR (Bone level, Roxolid), DENT (Superline), and NEO (Helix GM) are the safest implants because no aluminum was found in their chemical makeup [17]. To confirm the findings, additional research is required.

## Conclusion:

One weakness of the study was that it examined two distinct implant surfaces. The dental implants had different chemical surface features. Hence, no inferences about how representative these samples are of the manufacturer's output can be made with such a small sample size. Surface topography and roughness of the implants examined in this work will be measured quantitatively using various contact and non-contact profilometry methods due to the significance of surface morphology of implants for the osseointegration process.

## Figures and Tables

**Table 1:** Elemental composition on the top land area of single piece compressive implant (Sample A)

**Top Land Area**	
**Weight %**	**Element**
3.27	NaK
18.86	AlK
1.75	SiK
1.02	MoL
1	ClK
1.32	KK
1.23	CaK
71.55	TiK

**Table 2:** Elemental composition on the plain area of spiral implant (Sample B)

**Plain Area**	
**Weight %**	**Element**
1.16	MoL
18.34	CrK
2.25	MnK
66.6	FeK
0.84	CoK
7.66	NiK
3.14	TaL

**Table 3:** Elemental composition on the top land area of spiral implant (Sample B)

**Top Land Area**	
**Weight %**	**Element**
2.41	N K
19.9	O K
12.43	AlK
0.4	SiK
0.39	ClK
0.57	CaK
59.65	TiK
4.25	V K
